# USP5 facilitates non-small cell lung cancer progression through stabilization of PD-L1

**DOI:** 10.1038/s41419-021-04356-6

**Published:** 2021-11-05

**Authors:** Jinghua Pan, Yiting Qiao, Congcong Chen, Hongjing Zang, Xiaojing Zhang, Feng Qi, Cunjie Chang, Fan Yang, Mengqing Sun, Shengbin Lin, Quandong Tang, Lina Li, Menglan Wang, Minjie Wu, Yongzhu Liu, Caiyong Lai, Jianxiang Chen, Guo Chen

**Affiliations:** 1 Department of Gynecology, The Sixth Affiliated Hospital of Guangzhou Medical University, Qingyuan People’s Hospital, 511518 Guangdong, P.R. China; 2 Department of Medical Biochemistry, Urology and General Surgery, School of Medicine and The First Affiliated Hospital, Jinan University, 510632 Guangzhou, P. R. China; 3 Division of Hepatobiliary and Pancreatic Surgery, Department of Surgery, NHC Key Laboratory of Combined Multi-organ Transplantation, First Affiliated Hospital, Zhejiang University School of Medicine, 310003 Hangzhou, P. R. China; 4 Department of Pathology, The Second Xiangya Hospital, Central South University, 410011 Changsha, P.R. China; 5 College of Pharmacy, School of Medicine, Department of Hepatology, Institute of Hepatology and Metabolic Diseases, the Affiliated Hospital of Hangzhou Normal University, Key Laboratory of Elemene Class Anti-Cancer Chinese Medicines, Engineering Laboratory of Development and Application of Traditional Chinese Medicines, Collaborative Innovation Center of Traditional Chinese Medicines of Zhejiang Province, Hangzhou Normal University, 311121 Hangzhou, Zhejiang P.R. China; 6 Department of Pathophysiology, Shantou University Medical College, 515041 Shantou, Guangdong P.R. China; 7 School of Biopharmacy, China Pharmaceutical University, 211198 Nanjing, P.R. China

**Keywords:** Predictive markers, Non-small-cell lung cancer

## Abstract

PD-L1(CD274) is a well-known immunosuppressive molecule, which confers immunoescape features to cancer cells and has become one of the major targets in cancer immunotherapies. Understanding the regulatory mechanisms that control PD-L1 protein expression is important for guiding immune checkpoint blockade therapy. Here, we showed that ubiquitin specific peptidase 5 (USP5) was a novel PD-L1 deubiquitinase in non-small cell lung cancer (NSCLC) cells. USP5 directly interacted with PD-L1 and deubiquitinated PD-L1, therefore enhances PD-L1 protein stability. Meanwhile, USP5 protein levels were highly elevated and positively correlated to PD-L1 levels in NSCLC tissues, and were closely correlated with poor prognosis of these patients. In addition, knockdown of USP5 retarded tumor growth in the Lewis lung carcinoma mouse model. Thus, we identified that USP5 was a new regulator of PD-L1 and targeting USP5 is a promising strategy for cancer therapy.

## Introduction

Lung cancer is one of the most deadly cancers worldwide and the non-small cell lung cancer (NSCLC) accounts for 80–85% of all lung cancer cases [1]. Although the advances in chemotherapies and targeted therapies improved clinical outcomes, the overall 5-year survival rate for all NSCLC patients is still lower than 20%. Recently, targeting immune checkpoint mediated by programmed cell death protein 1 (PD-1) or its ligand PD-L1 achieves promising clinical response in numerous clinical studies, which opened a new era of NSCLC therapy [2–4]. However, only a limited subset of NSCLC patients could benefit from PD-1/PD-L1 immune checkpoint inhibition [5]. Therefore, the identification of novel molecular biomarker predicting therapeutic response are urgently needed.

PD-1 is an immunosuppressive receptor and is mainly expressed on activated T cells, B cells, and other immune cells [6]. As the ligand of PD-1, PD-L1 is highly expressed on various types of cancers, including NSCLC, and it plays an important roles in maintaining an immunosuppressive microenvironment to protect tumor cells from destruction by the immune system [7]. Therefore, blocking PD-1/PD-L1 signaling can elicit effective immune response against cancers. However, PD-L1 are expressed scatteredly among NSCLC tissues and a series of clinical and pre-clinical studies show that the therapeutic efficiency of PD-1/PD-L1 blockade is highly correlated to its PD-L1 expression [8]. The overall response rate (ORR) is 45.2%, 16.5%, and 10.7%, respectively for high, medium and low PD-L1 expression of NSCLC [9]. Thus, PD-L1 expression on NSCLC is considered as a predictive factor PD-1/PD-L1 inhibitor mediated immunotherapy and investigation of the regulatory mechanism for PD-L1 expression is valuable for directing future NSCLC immune therapy.

PD-L1 expression is regulated at transcriptional, translational, post-translational and epigenetic levels. Several transcription factors including STAT3, HIF-1α, NF-κB, and Myc have been reported to drive PD-L1 expression [10, 11]. Promoter methylation has been also reported be involved in regulation of PD-L1 expression epigenetically and treatment of DNA hypomethylating agent azacytidine can upregulate PD-L1 expression in NSCLC cells [12, 13]. More importantly, PD-L1 is regulated by ubiquitin-dependent proteasome degradation and several E3 ubiquitin ligase have been reported, such as Cullin3^SPOP^ and Cbl-b [14, 15]. On the contrary, COP9 signalosome 5 (CSN5) was identified as a PD-L1 deubiquitinase, which could antagonize the biological function of polyubiquitination and stabilizes PD-L1 in response to TNF-α stimulation [16]. Here, we demonstrated that ubiquitinspecific peptidase 5 (USP5) is an endogenous PD-L1 deubiquitinase in NSCLC cells. USP5 interacted with and stabilized PD-L1. Physiologically, USP5 is overexpressed in NSCLC tissues and its expression is highly associated with poor overall survival in NSCLC patients.

## Methods

### Materials

Human USP5 siRNA (L-006095-00) were obtained from Dharmacon (Lafayette, CO, USA), USP5 shRNA in pLKO.1 vector targeting mouse USP5 were purchased from Sigma (USP5 shRNA: TRCN0000030737, USP5 shRNA-B: TRCN0000030738) (St. Louis, MO, USA). Recombinant PD-L1 protein (NBP1-98984) was purchased from Novus Biologicals (Centennial, CO, USA). Flag-PD-L1 and myc-USP5 plasmids were obtained from GenScript (Piscataway, NJ). Anti-USP5 (sc-390943), anti-HA (sc-57592), anti-β-Actin (sc-47778) and anti-Flag (sc-807) antibodies were purchased from Santa Cruz (Santa Cruz, CA). Anti-PD-L1 antibody was purchased from Cell Signaling Technology (Danvers, MA). Mouse PD-L1 cDNA was purchased from Sino Biological Inc. (Beijing, China).

### Cell culture and transfection

H1299, H460, and LLC cells were obtained from American Type Culture Collection (ATCC) and cultured in RPMI-1640 medium supplemented with 10% fetal bovine serum (FBS; ExCell Bio, Shanghai, China). These cell lines were tested for mycoplasma contamination. No further authentication for these cell lines was carried out by authors. Cell transfection was performed using Exfect 2000 transfection reagent (Vazyme, NJ, China) according to manufacturer’s instruction.

### Lentivirus and stable cell line generation

HEK293FT cells were co-transfected with mouse USP5 shRNA plasmid, psPAX2, and pCMV-VSV-G and the supernatant containing lentivirus particles were collected at 48 h after transfection. For generation of cell line stably expressing USP5 shRNA, LLC cells were grown at 50–80% confluence before adding the lentivirus, and then, treated with 1–3 µg/ml puromycin at 24 h after lentiviral infection. The stable clones were picked and the expression of USP5 was examined by western blot. To stably express mouse PD-L1(mPD-L1) in USP5 knockdown cells, flag tagged mPD-L1 in pLVX-Hyg vector was co-transfected with psPAX2 and pCMV-VSV-G to produce lentivirus particle. Then, the lentivirus carrying flag-mPD-L1 was infected with USP5 knockdown LLC cells expressing USP5 shRNA-B and the cells were selected in presence of 500 μg/ml hygromycin and 0.5 μg/ml puromycin. After selection, the expression of flag-mPD-L1 was examined by western blot.

### Recombinant protein preparation

WT or C335A mutant USP5 cDNA were cloned into pGEX-4T-1 to generate GST fusion proteins and expressed in *E. coli*. Recombinant BL21 *E. coli* were grown in LB media at 37 °C with shaking until OD600 reached 0.6 and then, 0.2 mM IPTG was added to induce the protein expression. Bacteria were then grown in 18 °C with shaking for 16 h before harvesting in PBS, and lysed by sonication. After centrifugation, supernatant was applied to glutathione sepharose column (GE healthcare, Chicago, IL, USA), followed by washing with PBS. The USP5 proteins were eluted with 10 mM glutathione in PBS, aliquoted and stored in −80 °C.

### Immunoprecipitation

Cells were suspended in EBC buffer (50 mM Tris-HCl pH = 7.6–8.0, 0.5% NP-40, 1 mM EDTA, and 1 mM β-mercaptoethanol) containing protease inhibitor cocktail (Sigma, MO, USA) and lysed by sonication, followed by centrifugation at 14,000 × *g* for 10 min. The supernatant was incubated with indicated antibodies and protein A/G sepharose beads overnight at 4 °C. After washing with EBC buffer three times, beads were boiled in 40 μl of 2× SDS-PAGE sample buffer and subsequently subjected to SDS-PAGE. Then, the western blot analysis was performed using specific primary antibodies and horseradish peroxidase (HRP) conjugated secondary antibodies, protein bands were visualized by chemiluminescence.

### Ubiquitination assay

The in vivo ubiquitination assay was performed as previously described [17]. H1299 cells were transfected with indicated constructs for 48 h and treated with 10 µM MG132 for 6 h before harvesting. Cells were collected in EBC buffer (50 mm Tris-HCl pH 7.6–8.0, 120 mm NaCl, 0.5% NP-40, 1 mm EDTA, 1 mm β-mercaptoethanol, 50 mm NaF, and 1 mmNa_3_VO_4_) supplemented with cocktail protease inhibitor (EMD Millipore, Burlington, MA) and lysed by sonication. After centrifugation at 15,000×*g* 15 min, the supernatants were incubated with specific antibody conjugated sepharose beads overnight at 4 °C with rotation. After extensive wash, the beads were boiled in 2× loading buffer for 5 min, and separated on SDS-PAGE, followed by western blot analysis with Ub. In vitro deubiquitination assay was performed according to previously reported [17], briefly, poly-HA-ubiquitinated Flag-PD-L1 were prepared by co-transfecting HEK293T cells with Flag-PD-L1 and HA-ubiquitin. Forty-eight hours later, cells were treated with 10 µM MG132 for 6 h before harvesting for subsequent immunoprecipitation by Flag, and the poly-HA-ubiquitinated Flag-PD-L1 was purified in high-salt buffer. Recombinant WT or C335A USP5proteins were then incubated in DUB buffer (50 mM Tris-HCl, pH 8.0, 50 mM NaCl, 1 mM EDTA, 10 mM DTT, and 5% glycerol) at 37 °C for indicated time. Thereactions were stopped by adding 2× SDS loading bufferand the boiling for 5 min. The ubiquitinated PD-L1 were then analyzed by western blot.

### T cell-mediated tumor cell killing

5 × 10^6^ of LLC cells were injected into female C57BL/6 mice, when tumor length reached about 10 mm, the splenocytes were obtained from tumor bearing mice. After removal of red blood cells by RBC lysis buffer (155 mM NH_4_Cl, 12 mM NaHCO_3_, 0.1 mM EDTA), the lymphocytes were stimulated with concanavalin A (conA) (5 µg/ml) for 48 h. Then, the lymphocytes were co-incubated with LLC cells transfected with control or USP5 siRNA for further 48 h. The culture supernatants were subjected to relative LDH release assay according to manufacturer’s instructions.

### Lewis lung carcinoma xenograft

Four-to-six-weeks-old C57BL/6 male mice were provided by Hangzhou Ziyuan Experimental Animal Technology Co., Ltd. (Hangzhou, China). All experiments were performed according to the Animal Research Committee of the first affiliated hospital, Zhejiang University. The mice were housed in a pathogen-free condition. Mice were randomly divided into distinct groups without any selective criteria and each group has five mice (*n* = 5). LLC cells expressing control (Ctrl), USP5 shRNA, or USP5 shRNA plus flag-mPD-L1 (2 × 10^6^/mice) were subcutaneously injected into the right flanks of C57BL/6 mice. Tumor growth was monitored and tumor volumes were measured by caliper once every 3 days and calculated with the formula: *V* = (*L* × *W*2)/2 (L, length; W, width) as described [18]. The investigator was not blinded during experiment or assessing the outcome.

### Immunohistochemical (IHC) staining

Formalin-fixed paraffin-embedded microarrays of human lung cancer tissues were obtained from US Biomax (BC041115d) for co-relation analysis and US Biomax (HLug-Ade150Sur-01) for survival analysis. IHC staining was performed using R.T.U. Vectastain Kit (Vector Laboratories) according to the manufacturer’s instructions. Primary antibodies including USP5 (1:200, IHC-00313, Bethyl Laboratories), Ki67 (1:500, #12202, Cell Signaling technology) and PD-L1 (1:100, #13684, Cell Signaling technology) were employed. The semiquantitative determination of IHC staining was measured using immunoscore based on both percentage of stained cells and staining intensity as described [19]. The intensity was defined as follows: 0, no appreciable staining; 1, weak intensity; 2, moderate intensity; 3, strong intensity; 4, very strong intensity. The immunoscore was calculated by multiplying the intensity by percentage of positive staining, producing a total range of 0–400.

### Statistical analysis

Data shown are from one representative experiment of at least three independent experiments and normally distributed data are expressed as mean ± SD. We chose the sample size that gives 80% power at the 0.05 level of significance (two-sided). The statistical significance of difference between groups was analyzed with two-sided Student’s *T*-test. The log rank test was used to test the differences in Kaplan–Meier survival assay. *P* < 0.05 was considered statistically significant.

## Results

### PD-L1 is physically associated with USP5

**Fig. 1:**
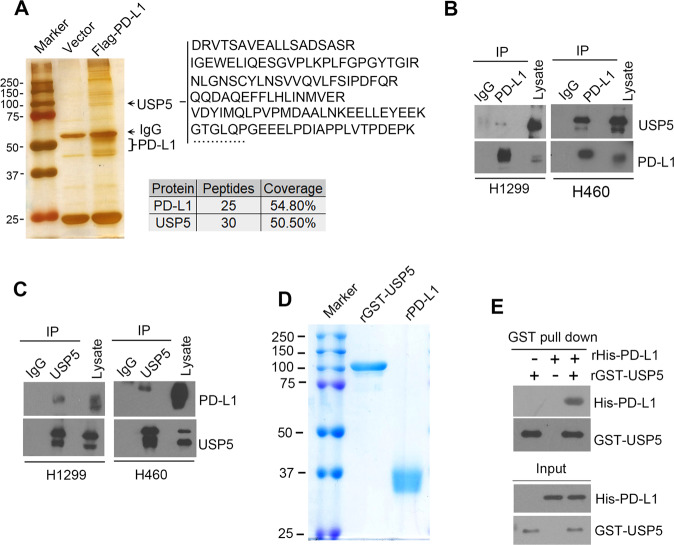
PD-L1 is physically associated with USP5. **A** Silver staining and mass spectrometry analysis of PD-L1 immunoprecipitation complex in H1299 cells. Representative peptides and coverage of PD-L1 and USP5 are shown. **B**, **C** Co-immunoprecipitation (co-IP) analysis from lysates derived from H1299 and H460 cells using anti-PD-L1 (**B**) or anti-USP5 (**C**) antibodies. **D** The coomassie blue staining of purified recombinant GST tagged USP5 and His tagged PD-L1 proteins. **E** In vitroGST-pull down analysis was performed using GST-USP5 and His-PD-L1.

In order to identify the deubiquitinase of PD-L1 in NSCLC cells, we performed immunoprecipitation (IP) assay using the cell lysates from Flag-PD-L1 transfected H1299 cells with anti-flag affinity beads. After extensively washing, the immunoprecipitation of the protein complexes was visualized by silver staining on SDS-PAGE and subjected to mass spectrometry analysis (Fig. 1A). The number of proteins including deubiquitinase USP5 was found to be associated with PD-L1 (Fig. 1A).

To validate the interaction between USP5 and PD-L1, we performed co-IP analysis in cell lysates derived from H1299 and H460 cells using anti-PD-L1 antibody. As shown in Fig. 1B, USP5 was detected to be associated with PD-L1 in both cell lines. On the contrast, we also observed PD-L1/USP5 interaction through co-IP assay with anti-USP5 antibody in indicated cells (Fig. 1C). To further test whether USP5 could directly binds to PD-L1, we prepared recombinant GST-USP5 (rUSP5) and recombinant His_6_-PD-L1 (rPD-L1) protein (Fig. 1D). Using these purified proteins, we demonstrated that USP5 directly interacts with PD-L1 through GST pull down assay (Fig. 1E).

### USP5 enhances PD-L1 protein stability

**Fig. 2:**
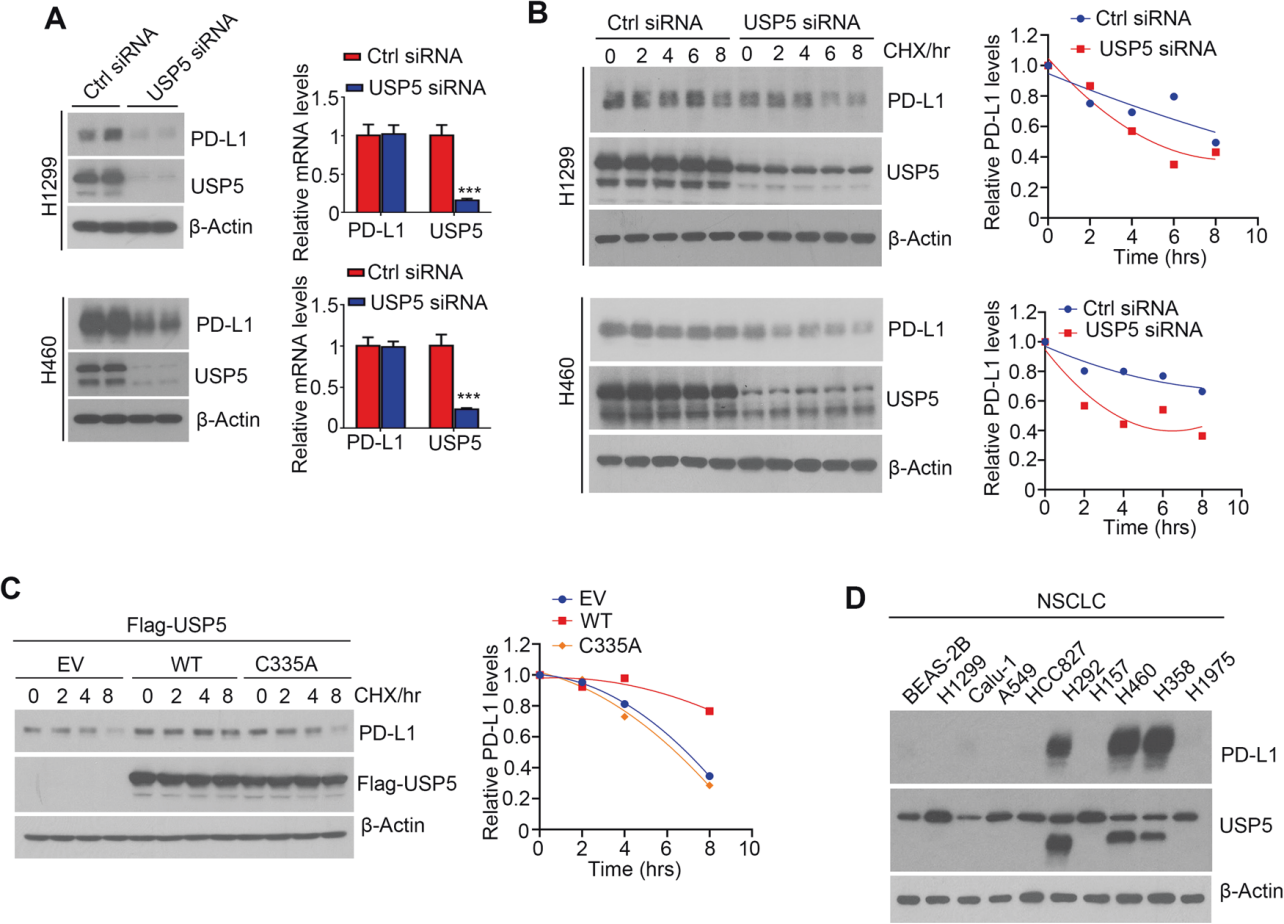
USP5 regulates PD-L1 stability. **A** H1299 and H460 cells weretransfected with non-targeting (ctrl) or USP5 siRNA, followed by western blot and qPCR analysis of PD-L1 protein and mRNA levels. **B** H1299 and H460 cells were transfected with non-targeting (ctrl) or USP5 siRNA, 48 h after transfection, cells were treated with 100 µg/ml cycloheximide (CHX) for the 2–8 h and followed by western blot analysis of indicated proteins. Representative western blots (left) and quantification of PD-L1 band intensities (right) were shown. **C** H1299 cells were transfected with empty vector (EV), WT, or C335A mutant flag-USP5, 48 h after transfection, cells were treated with 100 µg/ml cycloheximide (CHX) for the indicated time points and PD-L1 protein levels were checked by western blot (left). Right, PD-L1 protein level quantification was shown. **D** Protein levels of PD-L1 and USP5 were analyzed in different NSCLC cell lines by western blotting.

To address the functional significance of USP5/PD-L1 interaction, we examined the effect of USP5 knockdown on PD-L1 protein expression. The western blot analysis showed that USP5 knockdown decreased PD-L1 protein levels in H1299 and H460 cells (Fig. 2A). Meanwhile, knockdown of USP5 had no effects on PD-L1 mRNA levels (Fig. 2A). To test whether USP5 silence affects PD-L1 protein stability, H1299 or H460 cells transfected with control or USP5 siRNA were incubated with 50 µg/ml cycloheximide (CHX) and harvested at indicated time points, western blot assay showed that USP5 knockdown obviously decrease half-life of PD-L1 protein (Fig. 2B). In contrast, overexpression of WT USP5 could significantly increase PD-L1 half-life (Fig. 2C). However, overexpression of C335A USP5, the enzymatically inactive mutant, failed to prolong PD-L1 half-life (Fig. 2C), which indicates USP5 deubiquitinaseactivity is required for enhancing PD-L1 protein stability.

As an immunosuppressive molecule, PD-L1 is upregulated by pro-inflammatory cytokine TNF-α and genotoxic agents. Consistently, treatment of TNF-α and DNA damage agents including hydroxyurea (HU), aphidicolin (APH) and camptothecin (CPT) significantly increase PD-L1 protein levels, however, we did not observe increase of USP5 protein levels during these treatments (Fig. S1). We next evaluated the PD-L1 and USP5 protein levels in NSCLC cell lines, we found that PD-L1 expressions are divergent among NSCLC cells and PD-L1 is highly expressed in H292, H460 and H358 cells (Fig. 2D). Meanwhile, full length and a shorter cleaved form of USP5 were detected as previously shown [20], and high PD-L1 expressing cells contains relatively high total USP5 including full length and cleaved form (Fig. 2D).

### USP5 deubiquitinates PD-L1

**Fig. 3:**
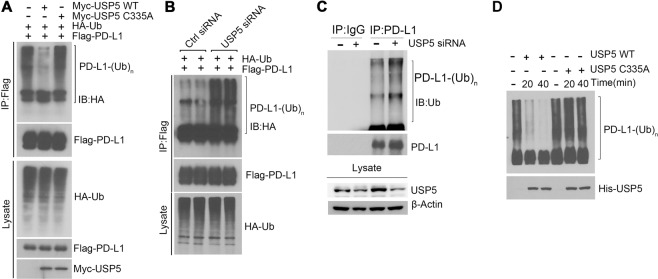
USP5 deubiquitinates PD-L1. **A** Immunoprecipitates and western blot analysis of cell lysates derived from H1299 cells transfected with HA-Ub, Flag-PD-L1, Myc-USP5 WT/C335A and treated with 5 µM MG132 for 6 h. **B** H1299 cells with or without USP5 knockdown by siRNA were co-transfected with HA-Ub and Flag-PD-L1, immunoprecipitation was performed using anti-FLAG affinity beads after 5 µM MG132 treatment for 6 h. Poly-ubiquitination of PD-L1 was analyzed by western blot with anti-HA antibodies. **C** H1299 cells were transfected with or without USP5 siRNA, 72 h after transfection, cells were lysed and subjected to anti-PD-L1 immunoprecipitation (IP), followed by analysis of PD-L1 ubiquitination using immunoblotting with anti-Ub(ubiquitin) antibody. **D** USP5 deubiquitinates polyubiquitination of PD-L1 in vitro. Flag-PD-L1 and HA-Ub were transfected in HEK293T cells. Poly-ubiquitinated PD-L1 was then immunoprecipitated with anti-Flag M2 beads and incubated with purified USP5-WT/C335A protein as indicated time. Lysates were analyzed by IB with indicated antibodies.

We next investigated whether USP5 mediated PD-L1 stabilization is a consequence of USP5 catalyzed deubiquitination of PD-L1. As expected, we found overexpression of wild-type (WT) USP5, but not catalytically inactive mutant USP5 (C335A) significantly reduced polyubiquitin chain linked with PD-L1 (Fig. 3A). Meanwhile, knockdown of endogenous USP5 enhanced PD-L1 ubiquitination (Fig. 3B). In addition, silence of USP5 also could increase endogenous PD-L1 ubiquitination level (Fig. 3C). To demonstrate that USP5 could directly deubiquitinate PD-L1, we performed the in vitro deubiquitination assays. As the result shown, poly-ubiquitin chain of PD-L1 was cleaved by WT USP5 within 40 m, however, C335A USP5 failed to deubiquitinate poly-ubiquitinated PD-L1 (Fig. 3D). These results confirm that USP5 deubiquitinates PD-L1 in vivo and in vitro.

### USP5 knockdown inhibits tumor growth through downregulating PD-L1

**Fig. 4:**
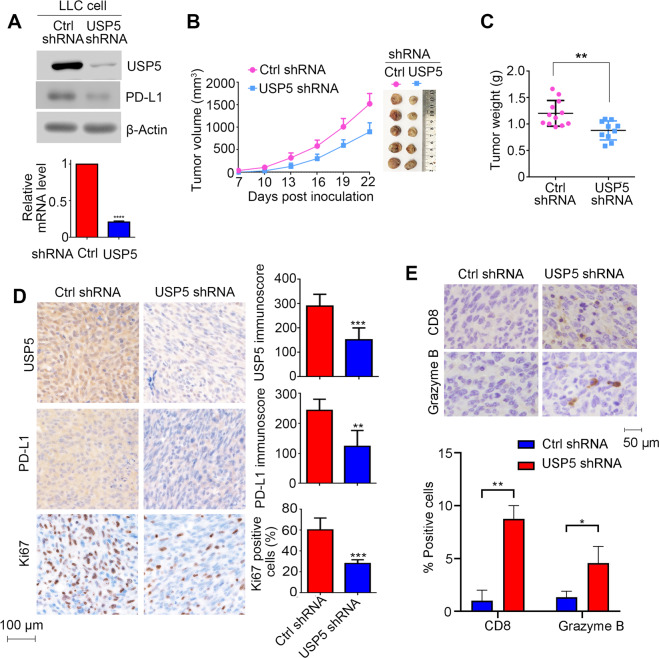
USP5 knockdown inhibits tumor growth in Lewis lung cancer xenografts. **A** Western blotting analysis of Lewis lung carcinoma (LLC) cells expressing control (ctrl) or USP5 shRNA. **B**, **C** LLC cells expressing ctrl or USP5 shRNA have been implanted into C57/BL6 mice, **B** Tumor growth curve and **C** tumor weights have been measured. **D** Immunohistochemistry (IHC) analysis of USP5, PD-L1, and Ki67 levels in LLC xenografts expressing ctrl or USP5 shRNA. Representative image (left) and quantification (right) of IHC stainig were shown. **E** IHC staining analysis of CD8 and Grazyme B positive cells in LLC xenografts expressing ctrl or USP5 shRNA. Data represents the mean ± SD, *n* = 5 per group. *P < 0.05, **P < 0.01, and ***P < 0.001.

To validate the role of USP5 in PD-L1 function, we first measured cell surface PD-L1 level upon USP5 depletion. As shown in Fig. S2, we observed that USP5 knockdown reduced membrane PD-L1 level. To further investigate the potential oncogenic roles of USP5-PD-L1 axis on lung cancer progression in vivo, we established mouse Lewis lung carcinoma cells (LLC) stably expressing control (Ctrl) or USP5 shRNA, in which the USP5 protein and mRNA were successfully knockdown (Fig. 4A). Colony formation analysis revealed that knockdown of USP5 had no effects on LLC cell proliferation in vitro (Fig. S3). Given that PD-L1 maintains immunosuppressive microenvironment to promote cancer progression. To explore the effects of USP5 on tumor growth, we implanted LLC cells with or without USP5 knockdown into immune competentC57BL/6 mice. In contrast with in vitro growth, we observed that USP5 knockdown significantly retarded tumor growth and reduced tumor weight compared with cells expressing Ctrl shRNA (Fig. 4B, C). USP5 protein levels in tumor tissues was validated by IHC, meanwhile, we also observed significant decrease of PD-L1 and Ki67 levels in USP5 knockdown tumors (Fig. 4D). In addition, we observed significant increase of CD8+ and Granzyme B+ positive cells infiltration in USP5 knockdown tumors (Fig. 4E), indicating USP5 knockdown could activate anti-tumor immune response.

**Fig. 5:**
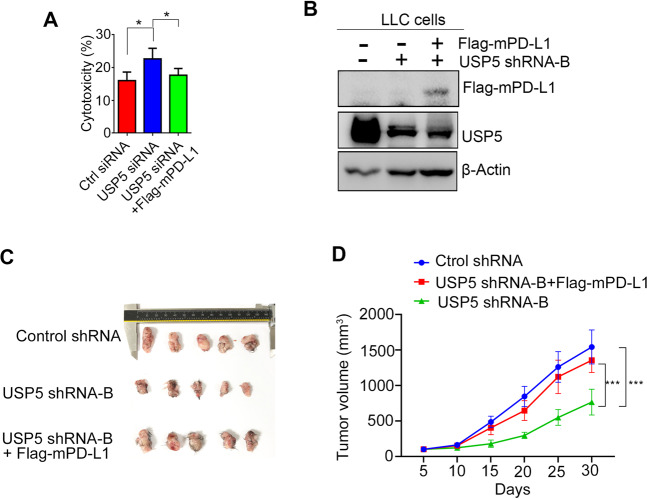
Ectopic expression of mouse PD-L1 (mPD-L1) could reverse USP5 knockdown induced tumor growth inhibition. **A** T-cell killing assay of LLC cells transfected with indicated USP5 siRNA or mouse Flag-PD-L1 (Flag-mPD-L1) was performed to as described in “Methods” section, and the cytotoxicity was evaluated by LDH release assay using the culture supernatant. **B** Western blot analysis of USP5 and PD-L1 expression in LLC cells transfected with indicated constructs. LLC cells expressing control, USP5 shRNA or USP5 shRNA plus Flag-mPD-L1 were implanted into right flank of C57BL6 mice. **C** Tumor image and tumor growth curve (**D**) was shown. *P < 0.05 and ***P < 0.001 by two-tailed *t*-test.

To further test whether USP5 knockdown caused LCC tumor growth inhibition was resulted from PD-L1downregulation, we next performed T cell-mediated cancer cell killing assay. T cells were isolated from mouse Lewis lung carcinoma (LLC)-bearing mice and then stimulated with concanavalin A before co-cultured with LLC cells transfected with USP5 siRNA or USP5 siRNA plus Flag tagged mouse PD-L1 (Flag-mPD-L1). As shown in Fig. 5A, we found that USP5 knockdown enhanced T cell-mediated cell killing in LLC cells, while, ectopic expression of PD-L1 rescued the effect. We then stably transfected Flag-mPD-L1 in USP5 silenced LLC cells carrying the other shRNA (shRNA-B) (Fig. 5B). As expected, overexpression of mPD-L1 significantly undermined tumor suppressive effect caused by USP5 knockdown (Fig. 5C, D). Taking together, these in vivo results confirmed that USP5 promotes lung cancer growth through regulating PD-L1 stability.

### USP5 expression is associated with poor prognosis of NSCLC

**Fig. 6:**
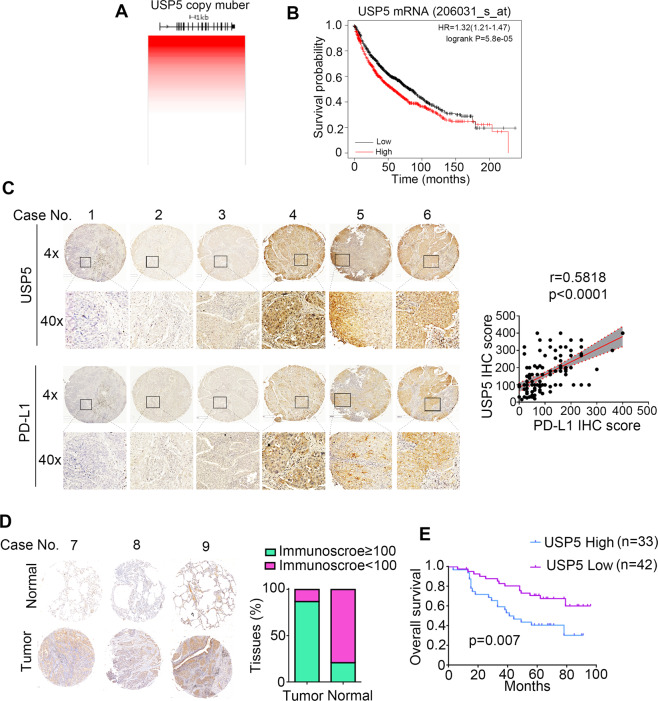
USP5 is overexpressed and highly associated poor prognosis in NSCLC patients. **A** Copy number analysis of *USP5* in TCGA lung adenocarcinoma. **B** Kaplan–Meier survival plot of USP5 mRNA in lung cancers using KM plotter (https://kmplot.com/). **C** IHC analysis of PD-L1 and USP5 protein levels in NSCLC tissues. The correlation between USP5 and PD-L1 in NSCLC patient tumors (*n* = 112) was explored using Pearson correlationanalysis. **D** IHC staining of USP5 was compared in NSCLC tissues and normal lung tissues. **E** Kaplan–Meier survival curve of USP5 protein expression in NSCLC patients, *n* = 74.

To determine the clinical relevance of the above findings in NSCLC patients, we first analyzed USP5 copy number of TCGA samples using UCSC-xena platform and found that USP5 is genomically amplified in NSCLC (Fig. 6A). The survival analysis by Kaplan–Meier plotter further showed that high USP5 mRNA is associated with poor survival in NSCLC patients (Fig. 6B). Meanwhile, immunohistochemical (IHC) staining analysis showed that USP5 protein levels are positively correlated with PD-L1 protein levels in NSCLC tissues (Fig. 6C), which is consistent that USP5 could stabilize PD-L1 through directly deubiquitinating its poly-ubiquitin chain. Furthermore, USP5 expression was upregulated in NSCLC tumor tissues compared with matched adjacent normal tissues (Fig. 6D). In addition, high level of USP5 is positively associated with poor prognosis (Fig. 6E). Collectively, these findings supported that USP5 is a modulator for PD-L1 protein expression and promotes NSCLC progression.

## Discussion

Accumulating evidences showing that PD-L1 expression on cancer cells can predict clinical prognosis and therapeutic efficiency of PD-1/PD-L1 blockade [8]. Post-translational modifications including glycosylation, phosphorylation, and polyubiquitination have been reported to be involved in the regulation of PD-L1 level [15, 21, 22]. Several E3 ligase (Cullin3-SPOP, c‐Cbl, and β-TrCP) and deubiquitinases (CSN5, USP9X, USP21, OTUB1, and USP22) have been reported to regulate PD-L1 protein degradation in cancer cells [14–16, 23–25], indicating posttranslational modification plays an important roles in regulation of PD-L1 stability. However, how the above deubiquitinases (CSN5, USP9X, USP21, OTUB1, and USP22) coordinatePD-L1 protein level in response to distinct tumor microenvironment signals remains unknown. Here, we identified that deubiquitinase USP5 is a PD-L1 binding partner and directly deubiquitinates and stabilizes PD-L1 in NSCLC cells. PD-L1 is often upregulated in response to inflammation and DNA damage, as evidenced by our observation that treatment of pro-inflammatory cytokine TNF-α and DNA-damaging agents significantly increase PD-L1 levels (Fig. S1) [16, 26]. Whereas, USP5 remains constant in the presence of such treatment, suggesting USP5 might not be involved in pro-inflammatory signaling or DNA damage induced PD-L1 upregulation. However, PD-L1 levels are highly correlated to USP5 in NSCLC cell lines and tissues, demonstrating USP5 is a deubiquitinase controls endogenous PD-L1 expression in NSCLC.

USP5, also named as isopeptidase T (IsoT), belongs to peptidase C19 family and prefer to cleave unanchored polyubiquitin chains [27], thus, it is crucial for free ubiquitin recycling. Besides its ubiquitin recycling function, USP5 is able to remove polyubiquitin chain on protein substrates and several proteins has been identified as USP5 substrates including FoxM1, c-Maf, and β-catenin [28–31]. It has been reported that knockdown of USP5 resulted in accumulation of unanchored polyubiquitin and activation of p53 pathway [32]. Therefore, USP5 is recognized as an oncogene for promoting tumorigenesis in various of cancers. Recently, USP5 has been show to promote epithelial–mesenchymal transition and metastasis through stabilizing SLUG protein level in hepatocellular carcinoma [29]. Consistently, we demonstrated here that USP5 level is elevated in human NSCLC cancersand its expression is highly correlated to poor prognosis in NSCLC patients. Consistent with previous reports [20], two cleaved isoforms have been observed in NSCLC cells, especially, in H292, H460, and H358 cells (Fig. 2D). Moreover, shorter form of USP5 is highly proportional to the PD-L1 protein levels in cancer cells (Fig. 2D), which indicates shorter form of USP5 might play more important role in regulating PD-L1 stability. In addition, USP5 protein level is positively proportionalto PD-L1 expression in NSCLC cancer tissues, indicating USP5 exerts oncogenic roles may partially through promoting tumor immune escape.

In conclusion, the present study demonstrated that USP5 is an endogenous deubiquitinaseof PD-L1 in NSCLC cells. USP5 increases PD-L1 protein level through removing its polyubiquitin chain and preventing from degradation. Therefore, USP5 could be considered as a novel biomarker predicting PD-L1 expression on NSCLC cells. In addition, targeting USP5 using shRNA-mediated knock down could reduce PD-L1 protein and triggers anti-tumor immune response.

## Supplementary information

supplementary information

## Data Availability

All data generated to support the conclusions are included either in an article or in the Supplementary Materials.
