# Supplementary material for: USP5 facilitates non-small cell lung cancer progression through stabilization of PD-L1
Source: Cell Death Dis. 2021 Nov 5;12(11):1051. doi: 10.1038/s41419-021-04356-6 (PMC8571306; doi:10.1038/s41419-021-04356-6)
Supplement: Supplementary file 1 — supplementary information [file 41419_2021_4356_MOESM1_ESM.docx]

USP5 facilitates non-small cell lung cancer progression through stabilization of PD-L1

Jinghua Pan^1,2#^, Yiting Qiao^3#^, Congcong Chen^2#^, Hongjing Zang^4#^, Xiaojing Zhang^1^, Qi, Feng^2^, Cunjie Chang^5^, Fan Yang^5^, Mengqing Sun^5^, Shengbin Lin^2^, Quandong Tang^6^, Lina Li^5^ , Menglan Wang^5^, Minjie Wu^5^, Yongzhu Liu^1^*,Caiyong Lai^2*^, Jianxiang Chen^5*^, Guo Chen^2,7*^.

1 Department of Gynecology, the Sixth Affiliated Hospital of Guangzhou Medical University, Qingyuan People’s Hospital, Guangdong 511518, P.R. China;

2 Department of Medical Biochemistry, Urology and General Surgery, School of Medicine and the First Affiliated Hospital, Jinan University, Guangzhou510632, P. R. China;

3 Division of Hepatobiliary and Pancreatic Surgery, Department of Surgery, NHC Key Laboratory of Combined Multi-organ Transplantation, First Affiliated Hospital, Zhejiang University School of Medicine, Hangzhou 310003, P. R. China.

4 Department of Pathology, The Second Xiangya Hospital, Central South University, Changsha 410011, P.R. China;

5 College of Pharmacy, School of Medicine; Department of Hepatology, Institute of Hepatology and Metabolic Diseases, the Affiliated Hospital of Hangzhou Normal University; Key Laboratory of Elemene Class Anti-Cancer Chinese Medicines; Engineering Laboratory of Development and Application of Traditional Chinese Medicines; Collaborative Innovation Center of Traditional Chinese Medicines of Zhejiang Province, Hangzhou Normal University, Hangzhou, Zhejiang 311121, China.

6 Department of Pathophysiology, Shantou University Medical College, Shantou, Guangdong 515041, P.R. China;

7 School of Biopharmacy, China Pharmaceutical University, Nanjing 211198, P.R. China;

# These authors contributed equally to this work

*To whom correspondence should be addressed:

Guo Chen, E-mail: gchen84@jnu.edu.cn

Jianxiang Chen, E-mail: [chenjx@hznu.edu.cn](mailto:chenjx@hznu.edu.cn)

Caiyong Lai, E-mail: lcy2015@jnu.edu.cn

Yongzhu Liu, E-mail: lyz_1995@yeah.net

**SUPPLEMENTARY FIGURES:**


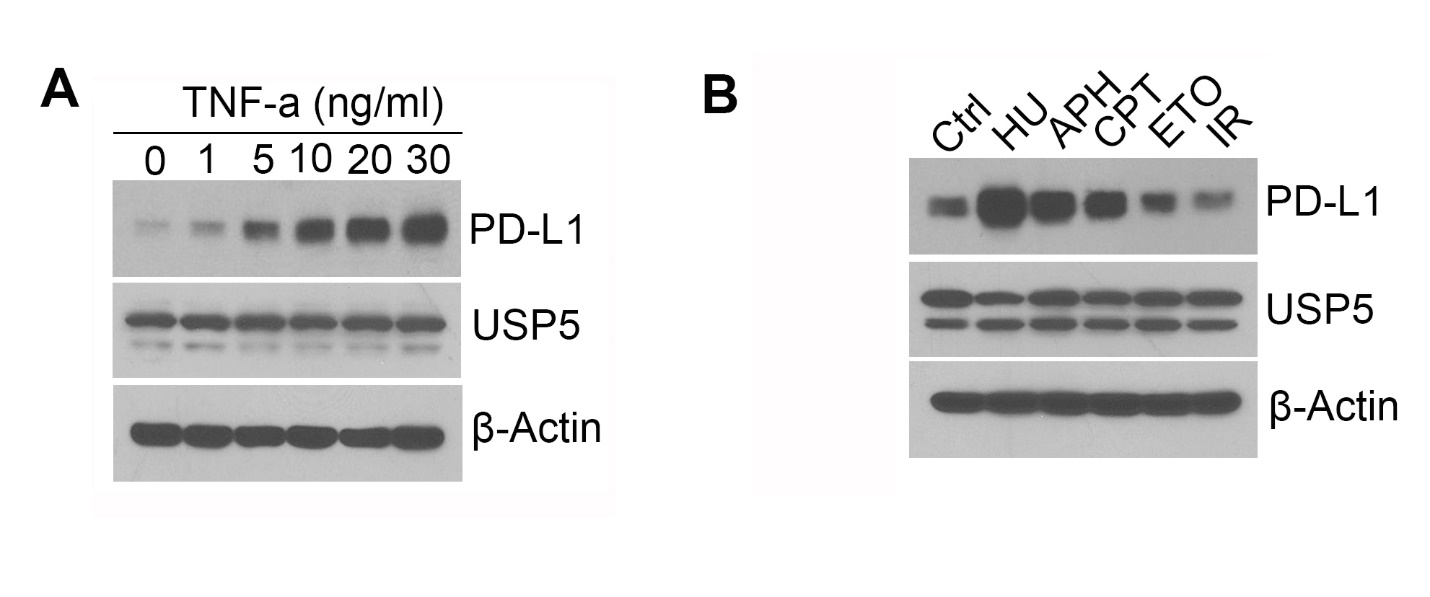


**Fig. S1.Inflammatory and DNA damage agents do not change USP5 protein levels.** (A) H1299 cells were treated with increasing concentrations of TNF-α, USP5 and PD-L1 protein levels were analyzed by Western blot. (B) H1299 cells were treated with 2mM hydroxyurea (HU, 12 hrs), 4 µM aphidicolin (APH, 12 hrs), 2 µM camptothecin (CPT, 6 hrs), 20 µM (etoposide, 12 hrs) and IR (10 Gy), USP5 and PD-L1 proteins levels were analyzed by western blot.


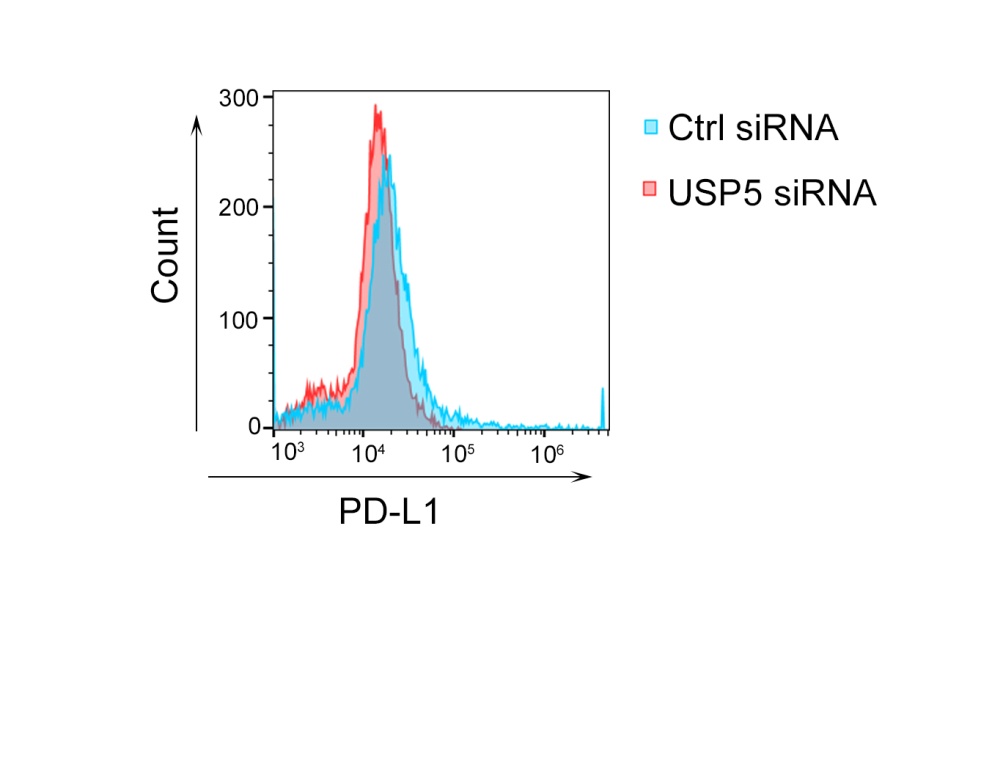


**Fig.S2 USP5 knockdown decrease PD-L1 cell surface expression.** Cell surface of PD-L1 was measured in H1299 cells transfected with control (ctrl) or USP5 siRNA using flow cytometry.


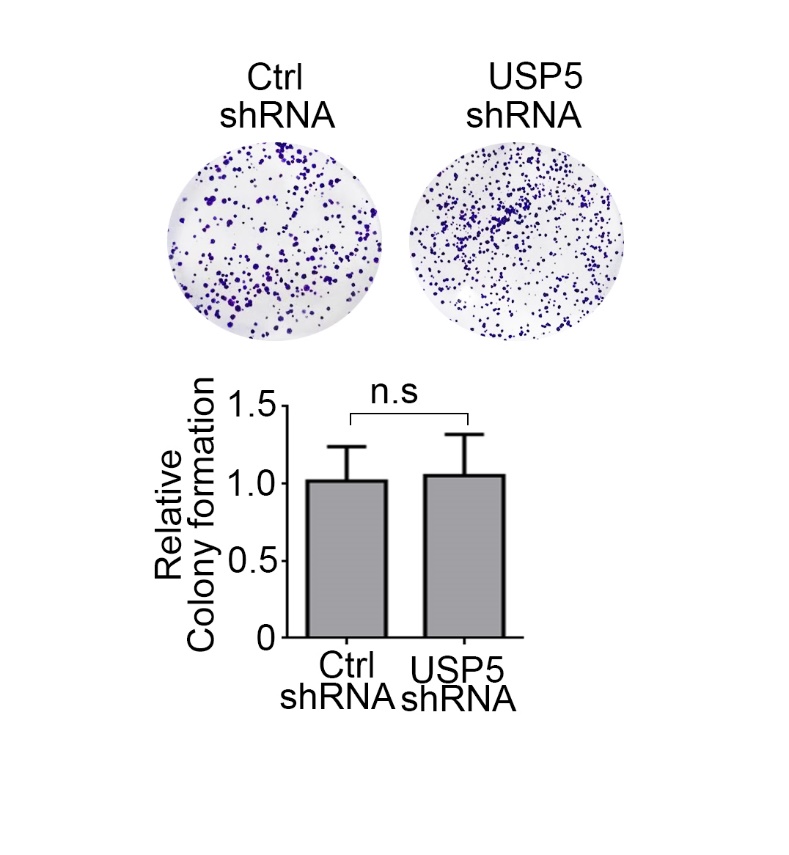


**Figure S3. Colony formation analysis of Lewis lung carcinoma (LLC) cells expressing control (Ctrl) or USP5 shRNA.**
